# PPARs and Metabolic Syndrome

**DOI:** 10.1155/2014/832606

**Published:** 2014-03-24

**Authors:** Lihong Chen, Zhanjun Jia, Guangrui Yang

**Affiliations:** ^1^The Institute for Translational Medicine and Therapeutics, University of Pennsylvania, Philadelphia, PA 19104, USA; ^2^Division of Nephrology and Hypertension, University of Utah, Salt Lake City, UT 84132, USA

Peroxisome proliferator-activated receptors (PPARs) exert versatile biological effects, notably in energy metabolism. During the last two decades, numerous studies have demonstrated that PPARs act as pivotal regulators of metabolic syndrome, a series of disorders in energy utilization and storage that are implicated with type 2 diabetes, diabetic nephropathy, and cardiovascular diseases, to mention a few. PPAR*α* and PPAR*γ* are the molecular targets of a number of marketed drugs for the treatment of these diseases, and accumulating evidence suggested PPAR*β*/*δ* as a potential therapeutic drug target as well. Although energy metabolism and metabolic syndrome are the most intensively studied domain of PPARs, it has not been addressed specifically in any issue of* PPAR Research* ever since its launch. Here, we gathered 3 reviews and 5 research articles that encompass metabolic syndrome and its complications.

M. Aprile et al. tackled the subject of PPAR*γ* and human adipogenesis in their research article. Rather than focusing on canonical PPAR*γ* transcripts, authors largely emphasized on the critical contribution of PPAR*γ* dominant negative isoforms to adipogenesis and their implied potential role in pathological conditions. In addition, a novel of PPAR*γ* dominant negative transcript, *γ*1ORF4, was first identified in this study. In regard to nonalcoholic fatty liver diseases, the hepatic expression of the metabolic syndrome, M. Sharif et al. conducted a thorough analysis of previously published data about the steatogenic role of PPAR*γ* and summarized two probable PPAR*γ* ligand-dependent toxicological modes of action: (i) activation of PPAR*γ* in hepatocytes and (ii) inhibition in adipocytes.

Two papers, one review and a research article, by Z. Jia and Y. Sun et al., appraised the role of PPAR*γ* in diabetic nephropathy (DN). Their comprehensive review summarized the limitations of traditional PPAR*γ* agonists, addressed the advantages of newly developed PPAR*γ* agonists, and rendered new insights into the therapeutic potential of PPAR*γ* agonists in the treatment of DN, while the research article suggested that a combination of PPAR*γ* agonists with COX-2/PGE2 inhibitors may be an alternative way of dealing with DN. In another research article, J. Jin et al. analyzed the correlation between PPAR gene polymorphisms and pediatric primary nephrotic syndrome (PNS) by comparing children with PNS against healthy subjects. They found that PPAR*γ* (Pro12Ala) and PGC-1*α* (Gly482Ser) polymorphisms are associated with abnormal insulin and triglyceride metabolism in pediatric PNS patients, suggesting that these polymorphisms may be relevant to the prognosis of this chronic disease.

The knowledge of the role of PPAR*α* in metabolic disorder-associated cardiovascular diseases was well recognized in this special issue. Z. Jia et al.'s research article asserted the involvement of HMGB1 (high mobility group box 1) in the protective effect of PPAR*α* in cardiac hypertrophy and provided a novel approach to study the pathogenesis of cardiac hypertrophy. Although most studies showed that PPAR*α* activation confers protection against atherogenesis, the intriguing possibility that PPAR*α* might foster atherogenesis is also considered. In this current issue, M. Vechoropoulos et al. found that PPAR*α* mediates the proatherogenic effect of chronic nitric oxide synthesis inhibition and this effect is independent of blood pressure and serum lipids alterations. These data further shaped the view that the role of PPAR*α* in atherosclerosis needs to be reevaluated.

Lastly, in the review article “*PPARs Integrate the Mammalian Clock and Energy Metabolism,*” we collected recent findings about the role of PPARs in biological clocks. This brand new function of PPARs bridges energy metabolism with circadian rhythm whose relationship has been known for long time, but not well understood. We summarized the circadian function of three PPAR subtypes one by one and concluded that the abnormality of PPARs and circadian rhythm could impinge on each other and thus leads to metabolic disorders. Further investigation of PPARs in this field will give us a new perspective on the therapeutic advances in the treatment of metabolic ailments.

In conclusion, this special issue is packed with intriguing novel breakthroughs and insights into PPARs and metabolic syndrome. We hope that these advances will generate more interest from the scientific community in better understanding of the role of PPARs in metabolic syndrome and associated complications.

*Lihong  Chen* *Lihong  Chen* *Zhanjun  Jia* *Zhanjun  Jia* *Guangrui  Yang* *Guangrui  Yang*

